# Supplementary material for: Optimized active layer morphology toward efficient and polymer batch insensitive organic solar cells
Source: Nat Commun. 2020 Jun 5;11:2855. doi: 10.1038/s41467-020-16621-x (PMC7275072; doi:10.1038/s41467-020-16621-x)
Supplement: Supplementary file 1 — Supplementary Information [file 41467_2020_16621_MOESM1_ESM.pdf]

SUPPLEMENTARY INFORMATION

Optimized Active Layer Morphology towards Efficient and Polymer Batch Insensitive Organic Solar Cells

Weng et al.

SUPPLEMENTARY METHODS

Characterizations of compounds

^1H NMR spectra were recorded on a Bruker AV-400 (400 MHz) NMR spectrometer. Chemical shifts were reported in parts per million (ppm, δ). ^1H NMR spectra were referenced to tetramethylsilane (0 ppm) for $CDCl_3$ as internal standard. Molecular weights of the polymers were obtained on a PL GPC 220 (Polymer Laboratories) at 160 °C using a calibration curve of polystyrene standards, with 1,2,4-trichlorobenezene as the eluent.

General information

Compounds M1 and T1-2Cl were synthesized according to the reported literatures[1,2]. Y6 was purchased from HYPER Inc. (Zhejiang, China). Other reagents and solvents were purchased from commercial sources and were used without further purification unless stated otherwise.

Synthesis of Random Copolymer PT2

0.1 mmol of M1, 0.07 mmol of T1-2Cl and 0.03 mmol of T1 were dissolve in a mixed solution of 4 mL toluene and 1 mL DMF. After $Pd(PPh_3)_4$ (1.5 mg) was added, the mixture was dealt with vacuo and nitrogen five times. Under nitrogen, the system was heated at 116 °C until just gelling (~45 min for the low molecular weight (LW) polymer). The mixture was poured into 20 mL methanol, and then the precipitation was dried in vacuo. Subsequently, the precipitation was dissolved in 30 mL $CHCl_3$, purified with a plug of silica gel, and precipitated in methanol again. Finally, the polymers were dried in vacuo (Supplementary Figure 1).

PT2 with higher molecular weights was synthesized by the method of prolonging the polymerization time with an energetic stir after gelling (~1 h for the medium e molecular weight (MW) polymer and ~4 h for the high molecular weight (HW) polymer). The polymer gel was poured into 20 mL methanol, and washed with methanol, acetone and dichloromethane in sequence. Finally, the polymers were dried

in vacuo. The ^1H NMR spectra of PT2 polymers with LW (45 k) and MW (57k) is presented in Supplementary Figure 2.

PT2 (LW): M_n = 45.3 kDa, PDI = 2.33. Elemental analysis: $C_{80}H_{96.6}Cl_{1.4}O_2S_6$: calcd. C 72.13, H 7.31; found: C 71.38, H 7.27.

PT2 (MW): M_n = 57.4 kDa, PDI = 2.05. Elemental analysis: $C_{80}H_{96.6}Cl_{1.4}O_2S_6$: calcd. C 72.13, H 7.31; found: C 71.46, H 7.22.

PT2 (HW): M_n = 91.2 kDa, PDI = 2.08. Elemental analysis: $C_{80}H_{96.6}Cl_{1.4}O_2S_6$: calcd. C 72.13, H 7.31; found: C 71.49, H 7.15.

Cyclic voltammogram curves of PT2 with different molecular weights.

The CV curves of three PT2 batches in 0.1 M tetrabutylammonium hexafluorophosphate (Bu4NPF6) acetonitrile solution are shown in Supplementary Figure 3. The highest occupied molecular orbital (HOMO)/lowest unoccupied molecular orbital (LUMO) of three PT2 batches were -5.57/-3.6 eV, -5.59/-3.61 eV and -5.54/-3.61 eV, respectively.

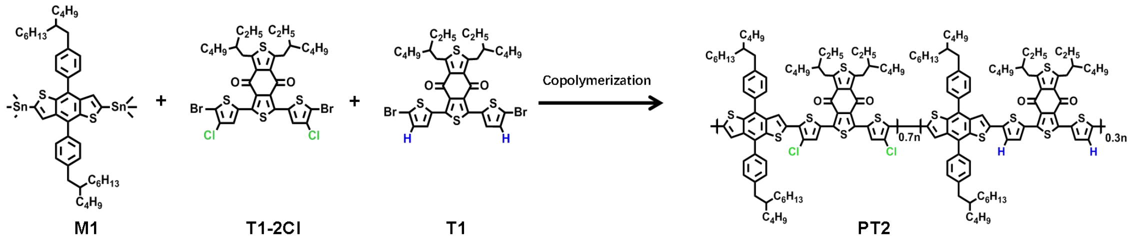

Supplementary Figure 1. Synthesis routes of PT2.

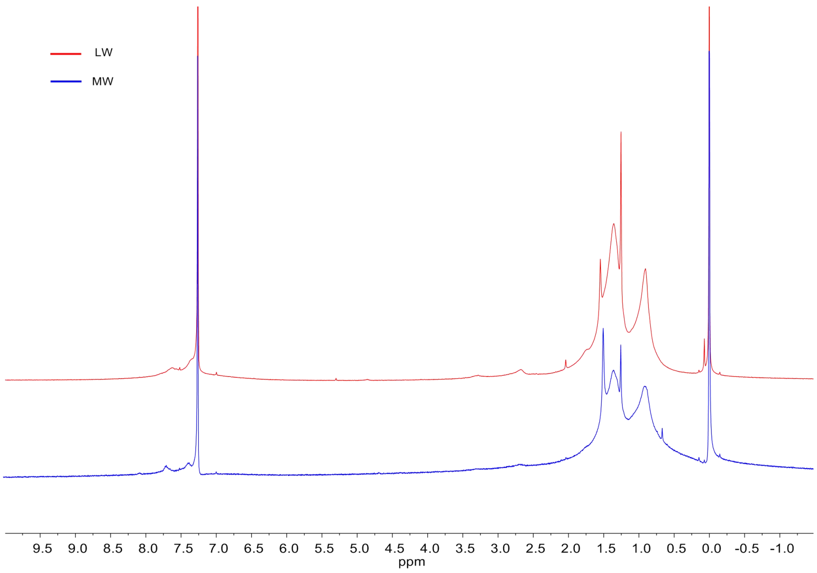

Supplementary Figure 2. ^{1}H NMR spectra of LW (45k) and MW (57k) PT2 polymers.

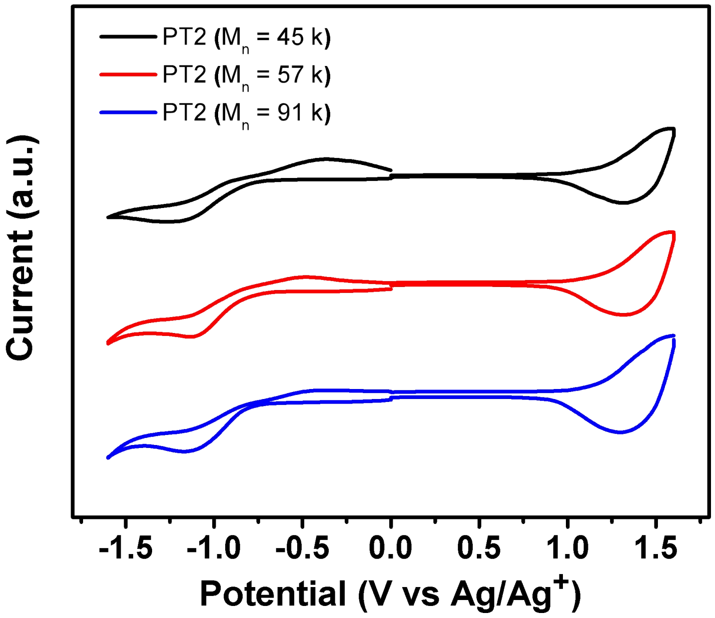

Supplementary Figure 3. Cyclic voltammogram curves of PT2 with different molecular weights.

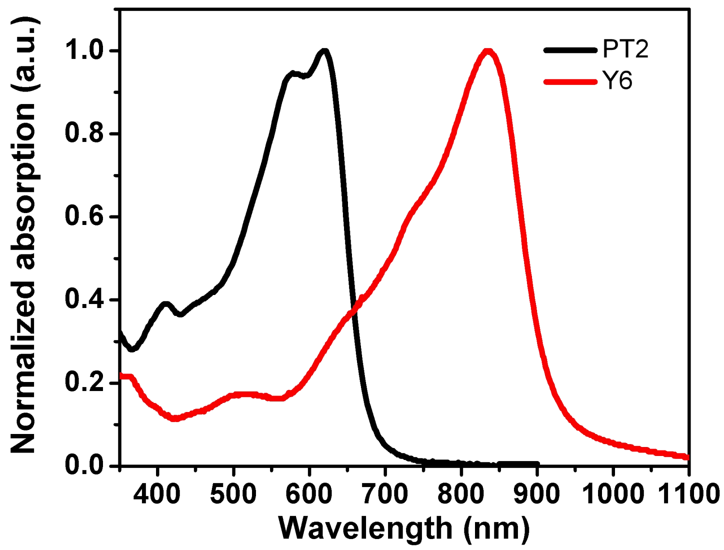

Supplementary Figure 4. Normalized UV-Vis absorption of PT2 and Y6 neat films.

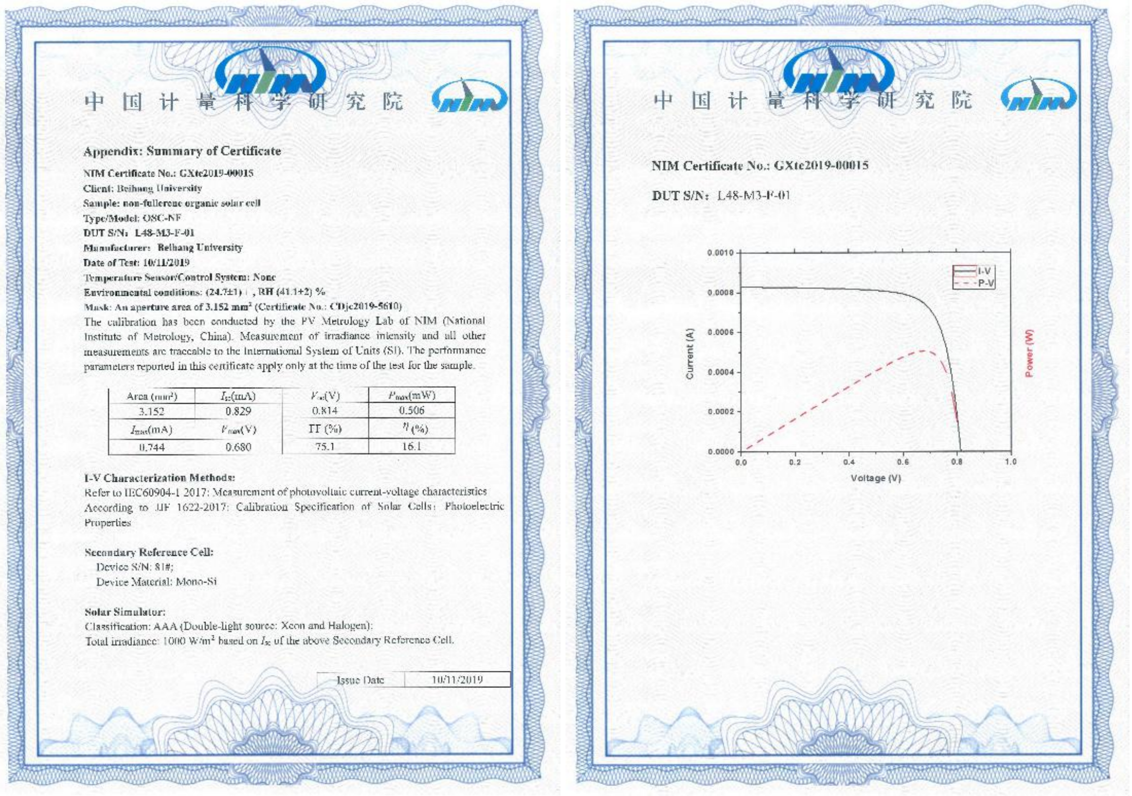

中国计量科学研究院

Appendix: Summary of Certificate

NIM Certificate No.: GXtc2019-00015
Client: Beihang University
Sample: non-fullerene organic solar cell
Type/Model: OSC-NF
DUT S/N: L48-M3-F-01
Manufacturer: Beihang University
Date of Test: 10/11/2019
Temperature Sensor/Control System: None
Environmental conditions: (24.7±1) [illegible], RH (41.1+2) %
Mask: An aperture area of 3.152 mm² (Certificate No.: CDjc2019-5610)

The calibration has been conducted by the PV Metrology Lab of NIM (National Institute of Metrology, China). Measurement of irradiance intensity and all other measurements are traceable to the International System of Units (SI). The performance parameters reported in this certificate apply only at the time of the test for the sample.

Area (mm^2)	I_{sc}(mA)	V_{oc}(V)	P_{max}(mW)
3.152	0.829	0.814	0.506
I_{max}(mA)	V_{max}(V)	FF (%)	η (%)
0.744	0.680	75.1	16.1

I-V Characterization Methods:
Refer to IEC60904-1 2017: Measurement of photovoltaic current-voltage characteristics
According to JJF 1622-2017: Calibration Specification of Solar Cells: Photoelectric Properties

Secondary Reference Cell:
Device S/N: 81#;
Device Material: Mono-Si

Solar Simulator:
Classification: AAA (Double-light source: Xeon and Halogen);
Total irradiance: 1000 W/m² based on I_{sc} of the above Secondary Reference Cell.

Issue Date	10/11/2019

中国计量科学研究院

NIM Certificate No.: GXtc2019-00015

DUT S/N: L48-M3-F-01

Supplementary Figure 5. Certification of the photovoltaic efficiency of the optimum SD device.

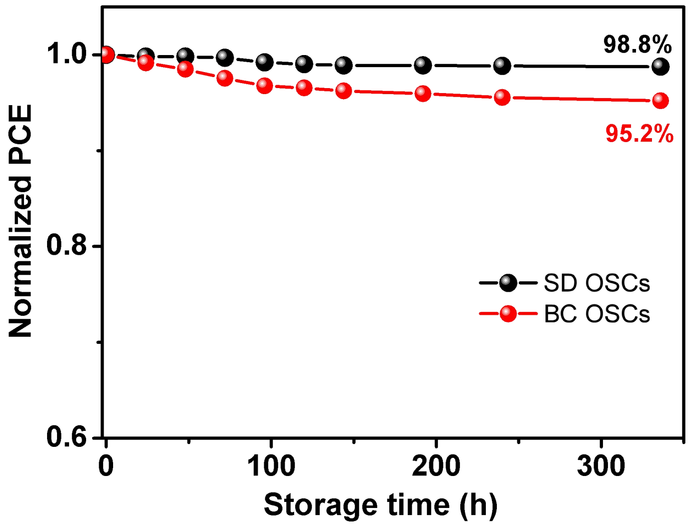

Supplementary Figure 6. Air stability tests of the encapsulated SD and BC OSCs. Source data are provided as a Source Data file.

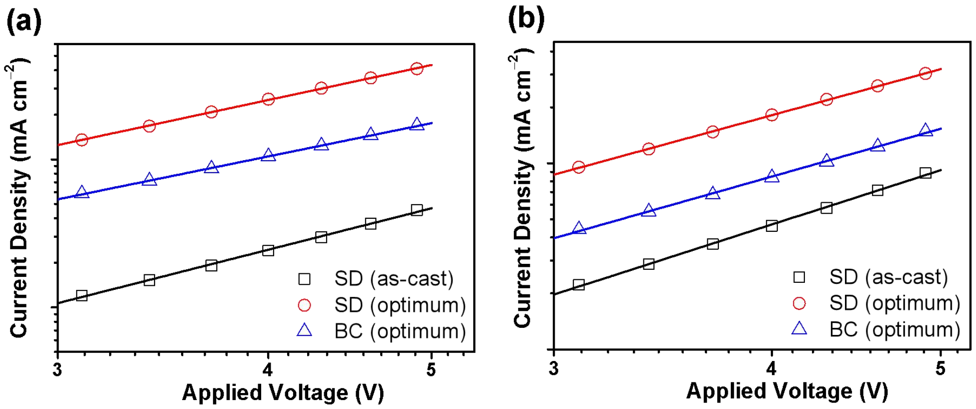

Supplementary Fig 7. a Hole-only and **b** electron-only mobilities of the blend films with different processing conditions.

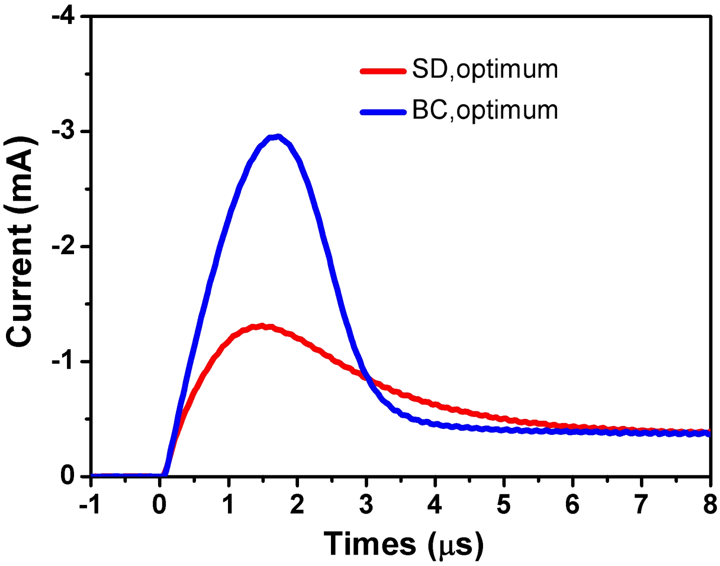

Supplementary Figure 8. Photo-CELIV-basic curves of the optimum SD and BC devices based on PT2:Y6 blend (M_n = 57k).

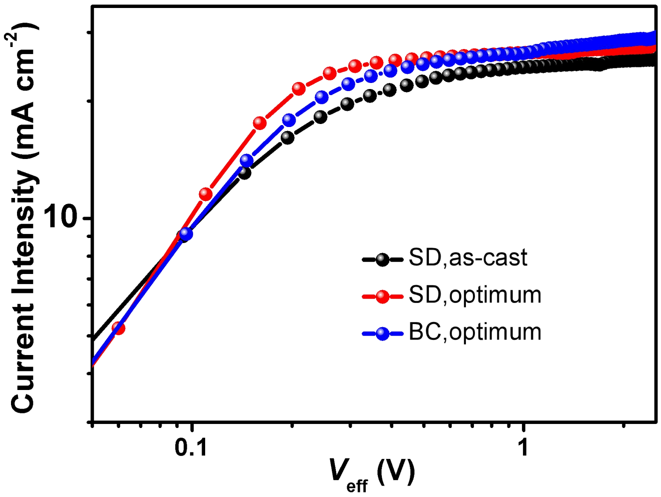

Supplementary Figure 9. Characteristics of the photocurrent density versus effective voltage for SD and BC OSCs.

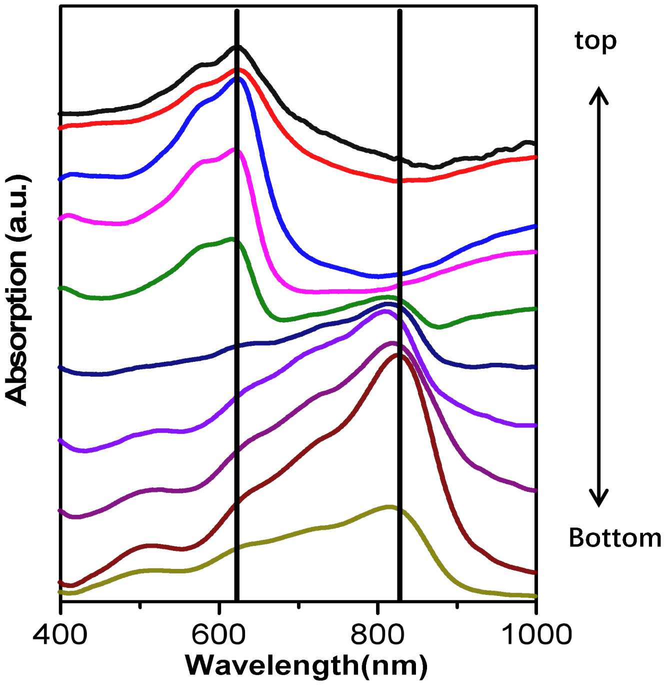

Supplementary Figure 10. Film-depth-dependent light absorption spectroscopy of layer-by-layer film deposited on the PEDOT:PSS/ITO, showing a well-defined two-layer architecture with PT2 and Y6 as top and bottom layer respectively. The solid lines are guides to eyes to shown the absorption peaks of donor and acceptor.

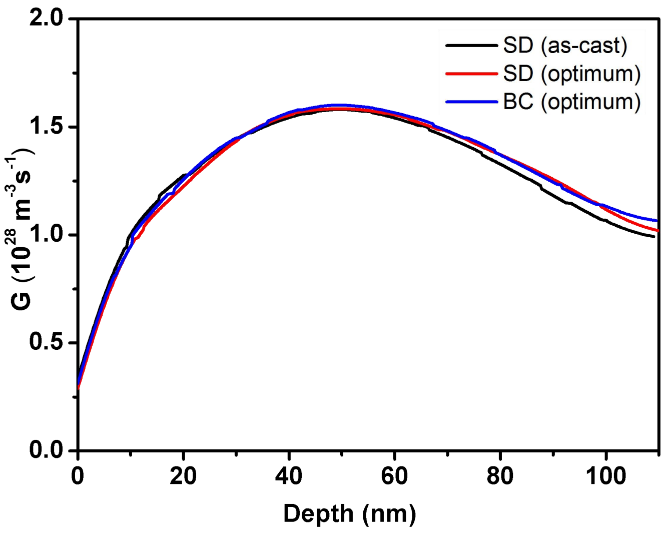

Supplementary Figure 11. Calculated exciton generation contours as simulated from the measured film-depth-dependent light absorption spectra.

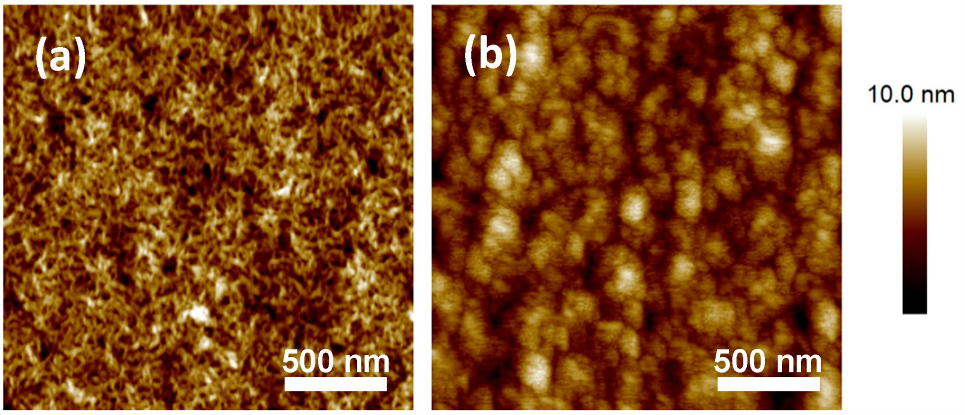

Supplementary Figure 12. AFM topography images of **a** PT2 neat film, and **b** Y6 neat film.

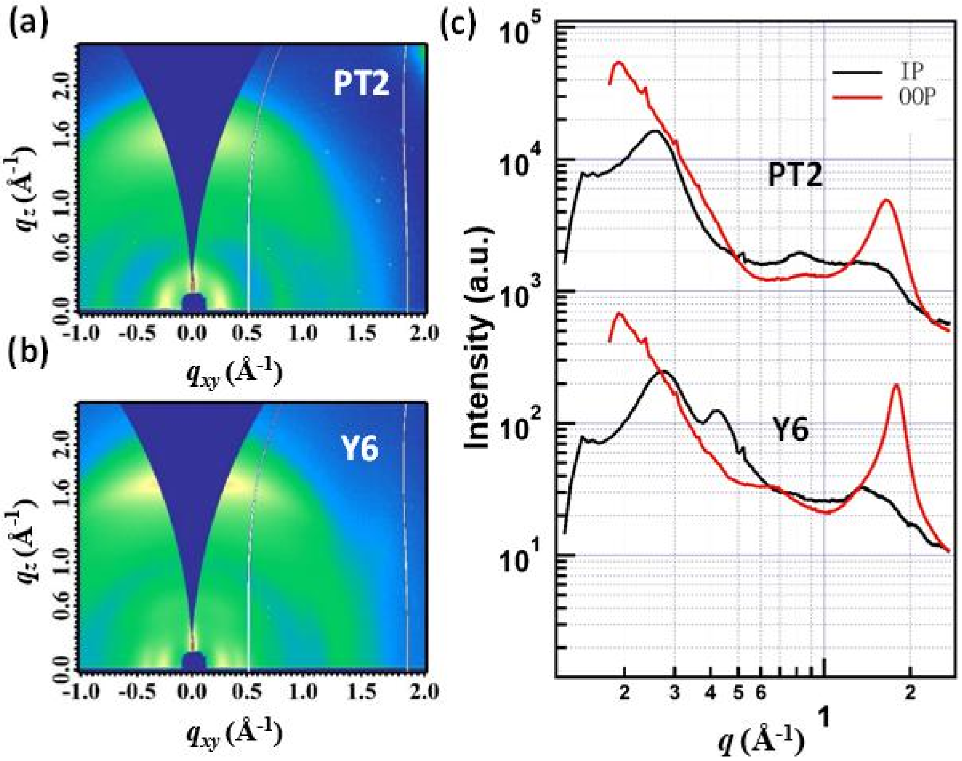

Supplementary Figure 13. a,b 2D GIWAXS diffraction patterns of PT2 and Y6 neat films. **c** In-plane (black lines) and out-of-plane (red lines) line-cut profiles of the 2D GIWAXS data.

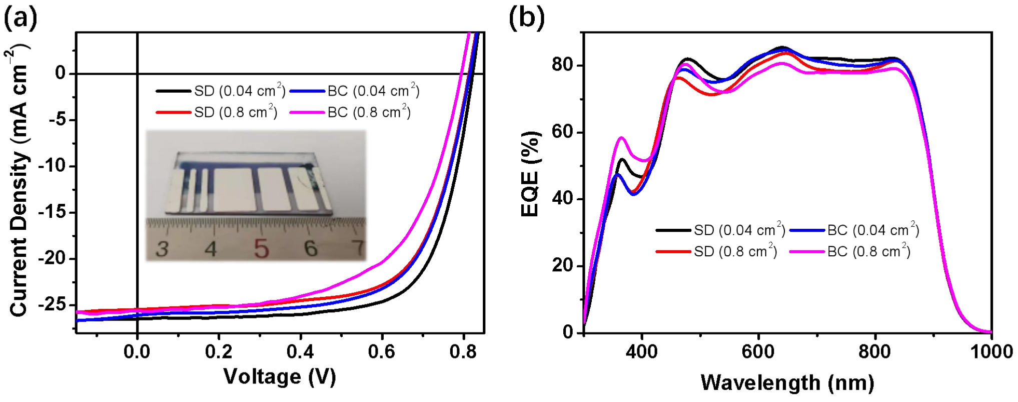

Supplementary Figure 14. a *J–V* curves of PT2:Y6 OSCs based on slot-die printed films with different device areas, and **b** the corresponding EQE spectra. The inset is a photograph of the real slot-die printed device.

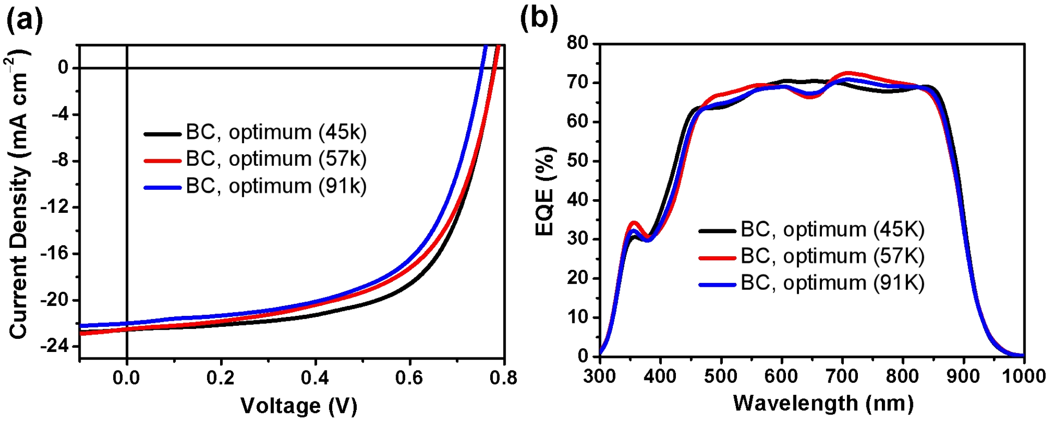

Supplementary Figure 15. **a** *J–V* characteristics of BC OSCs (PT2:Y6) fabricated with different PT2 batches under constant incident light intensity (AM 1.5G, 100 mW cm^{-2}), and **b** the corresponding EQE spectra. Chlorobenzene was used as the host solvent.

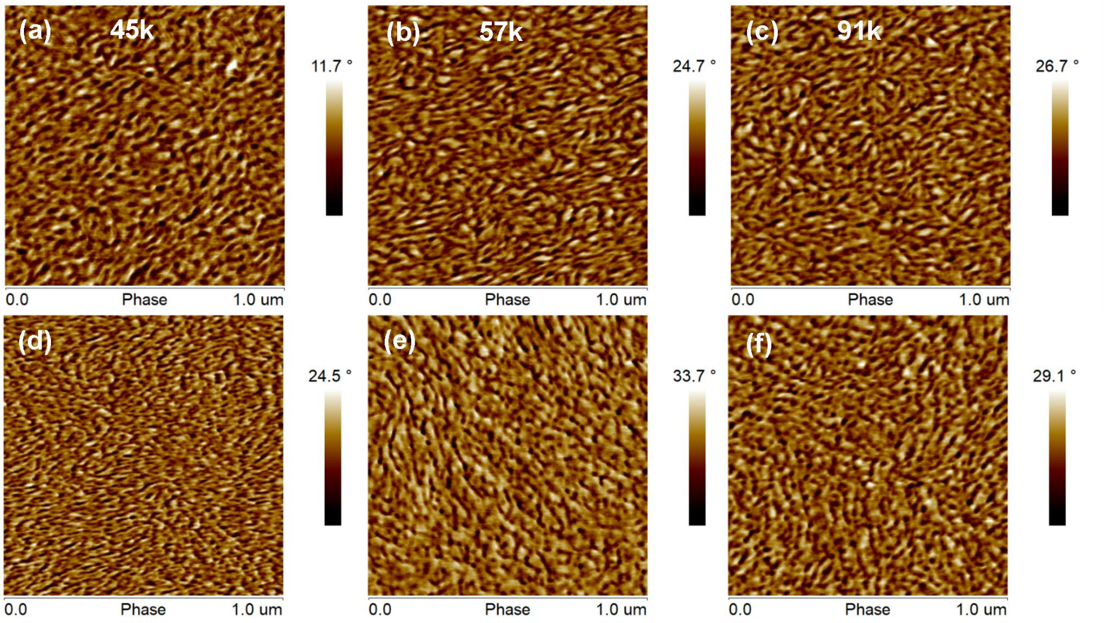

Supplementary Figure 16. AFM phase images of PT2 films. **a,d** PT2 films (M_n = 45k) without and with CF/DIO washing. **b,e** PT2 films (M_n = 57k) without and with CF/DIO washing. **c,f** PT2 films (M_n = 91k) without and with CF/DIO washing.

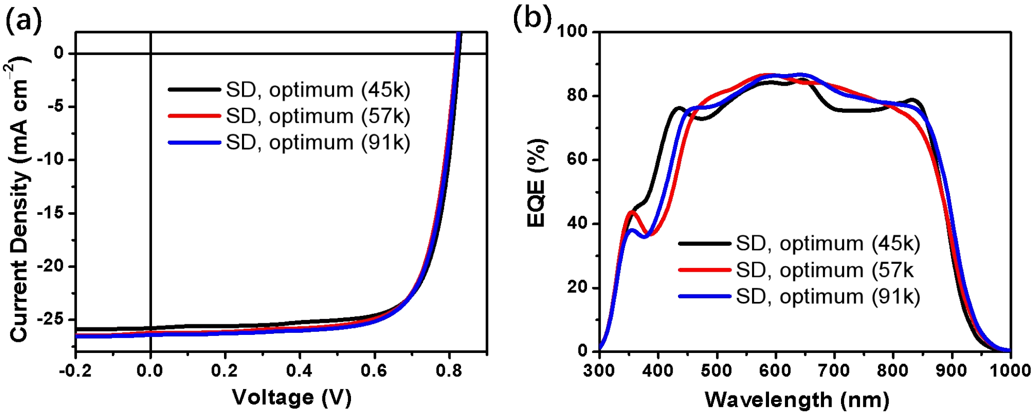

Supplementary Figure 17. a *J–V* characteristics of SD OSCs (PT2:Y6) fabricated with different PT2 batches under constant incident light intensity (AM 1.5G, 100 mW cm^{-2}), and **b** the corresponding EQE spectra. The PT2 sublayer was washed with CF/DIO solvent (1% DIO).

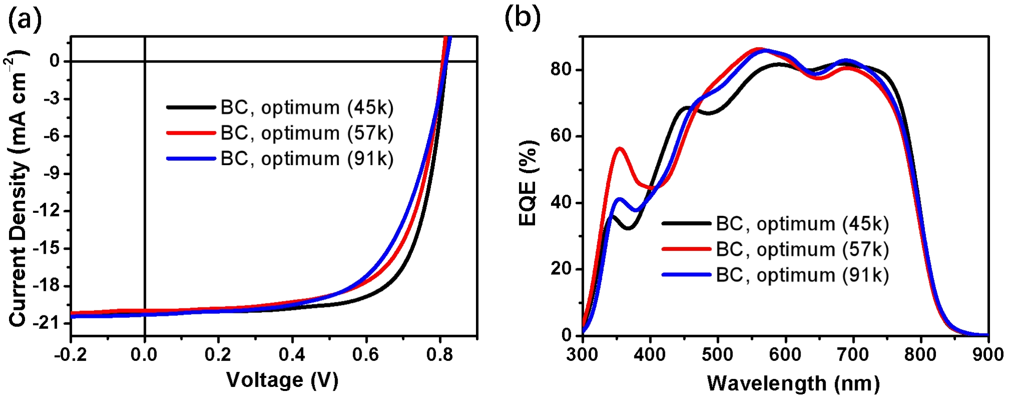

Supplementary Figure 18. a *J–V* characteristics of BC OSCs (PT2:IT-4F) fabricated with different PT2 batches under constant incident light intensity (AM 1.5G, 100 mW cm^{-2}), and **b** the corresponding EQE spectra. Chloroform was used as the host solvent.

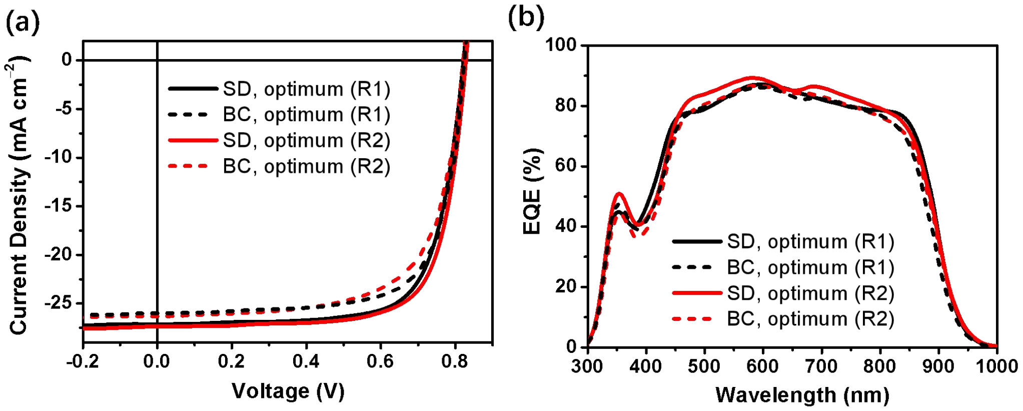

Supplementary Figure 19. a *J*–*V* characteristics of SD and BC OSCs (PT2:Y6) fabricated with two random PT2 batches under constant incident light intensity (AM 1.5G, 100 mW cm^{-2}), and **b** the corresponding EQE spectra.

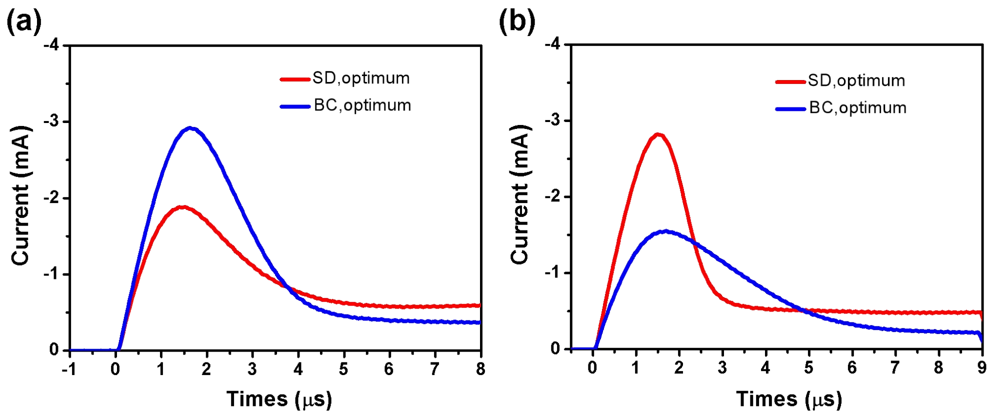

Supplementary Figure 20. Photo-CELIV-basic curves of SD and BC devices based on different PT2 batches. **a** PT2:Y6 (M_n = 45k) and **b** PT2:Y6 (M_n = 91k)

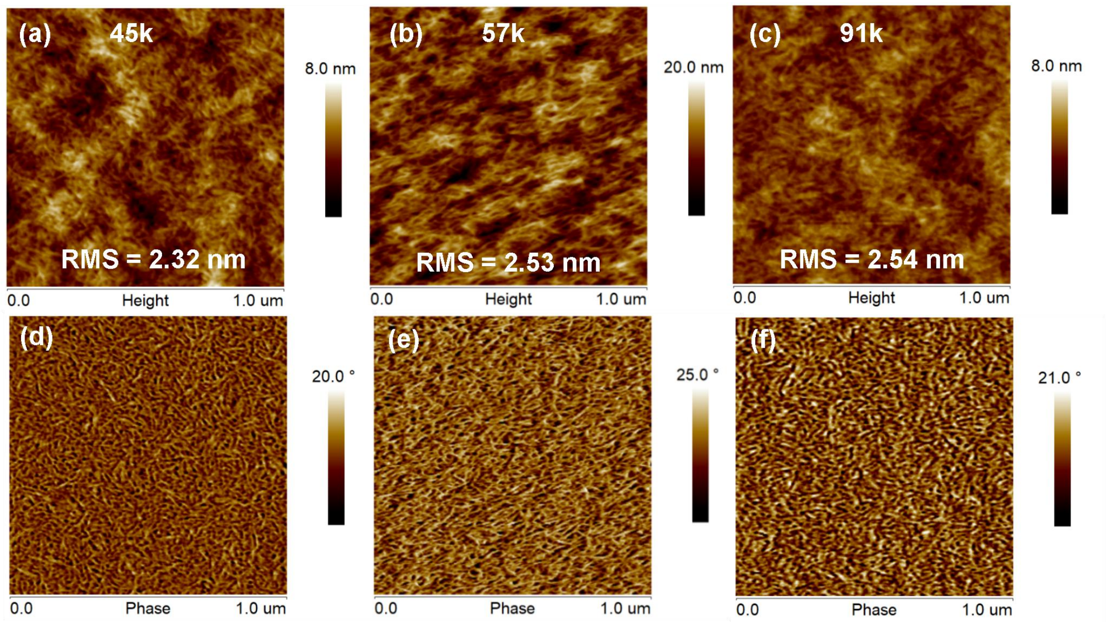

Supplementary Figure 21. AFM topography images of SD films fabricated with different PT2 batches. **a,d** PT2 (45k):Y6. **b,e** PT2 (57k):Y6. **c,f** PT2 (91k):Y6. PT2 and Y6 were processed from chlorobenzene and chloroform, respectively.

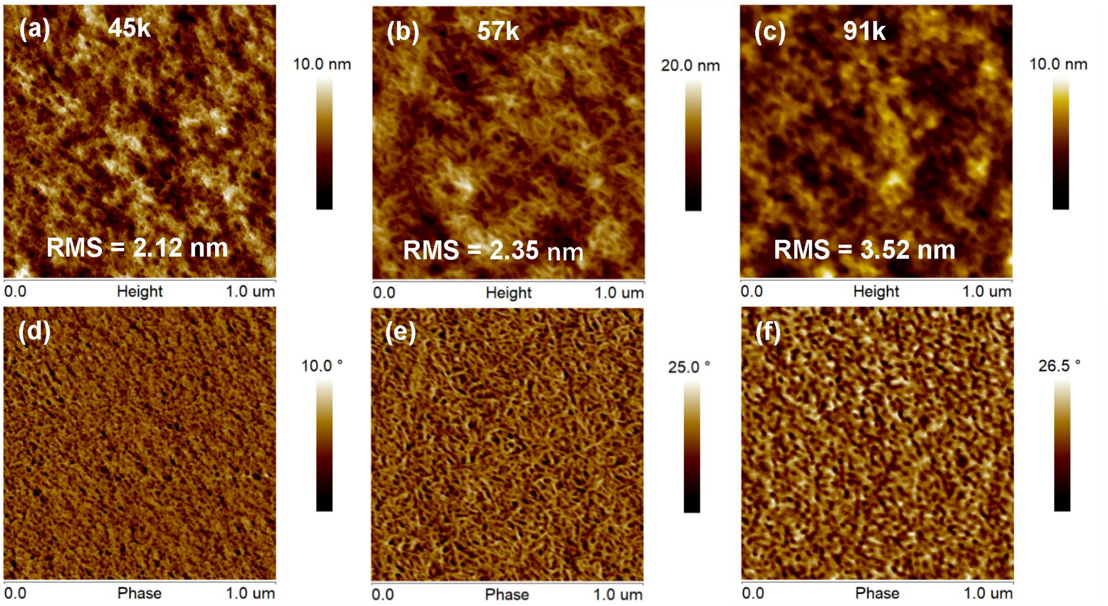

Supplementary Figure 22. AFM topography images of BC films fabricated with different PT2 batches. **a,d** PT2 (45k):Y6. **b,e** PT2 (57k):Y6. **c,f** PT2 (91k):Y6. Chloroform was used as the host solvent.

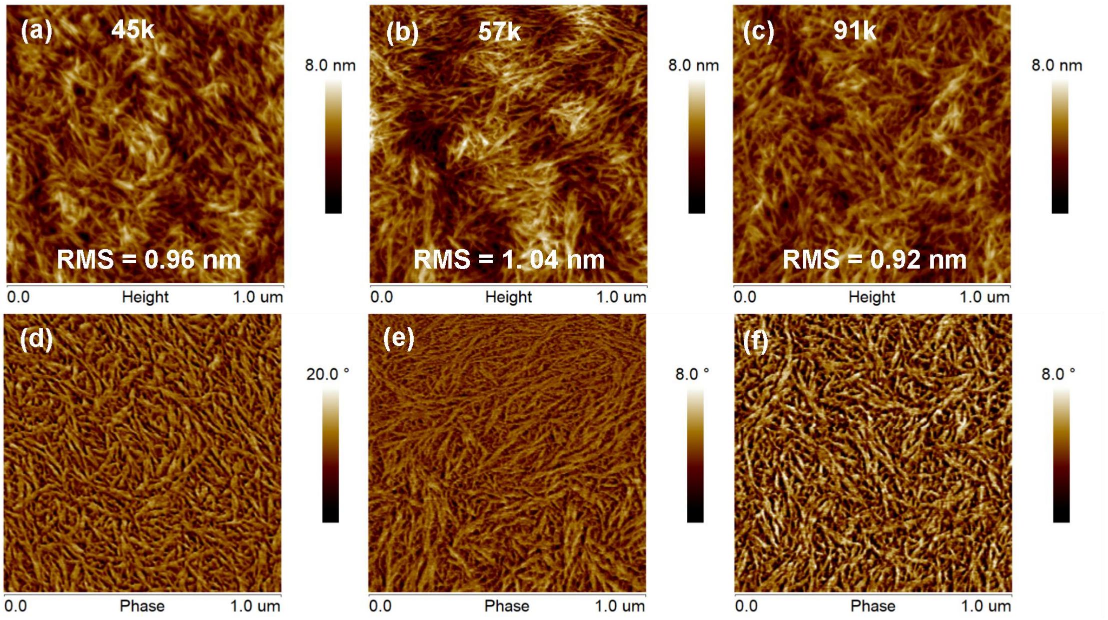

Supplementary Figure 23. AFM topography images of SD films fabricated with different PT2 batches. **a,d** PT2 (45k):IT-4F. **b,e** PT2 (57k):IT-4F. **c,f** PT2 (91k):IT-4F. The PT2 and IT-4F were processed from chlorobenzene and chloroform, respectively.

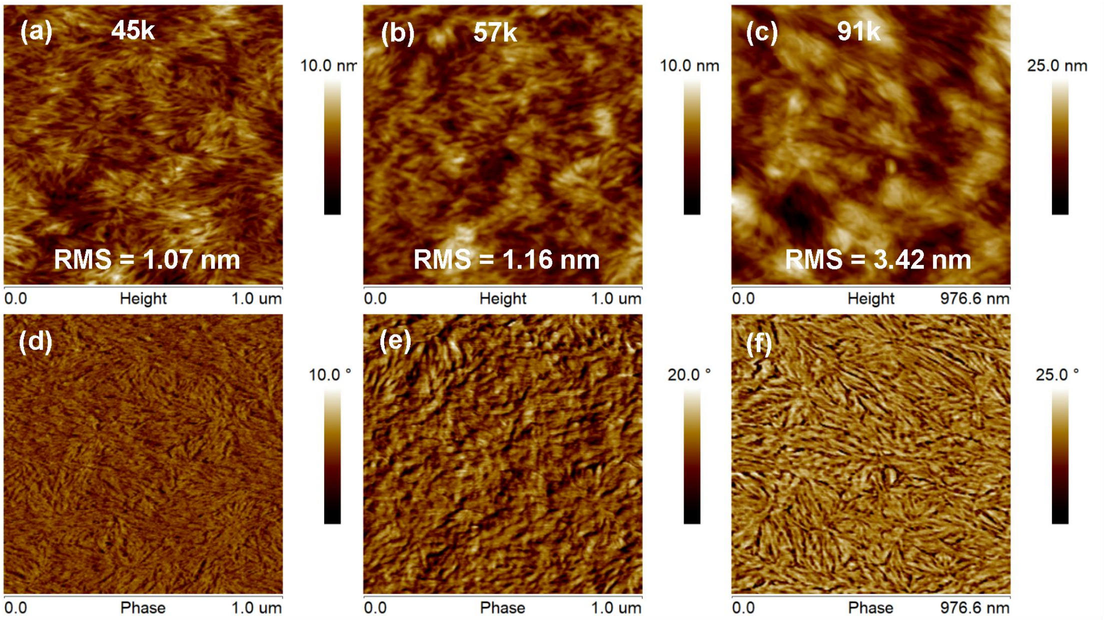

Supplementary Figure 24. AFM topography images of BC films fabricated with different PT2 batches. **a,d** PT2 (45k):IT-4F. **b,e** PT2 (57k):IT-4F. **c,f** PT2 (91k):IT-4F. Chlorobenzene was used as the host solvent.

Supplementary Table 1. Devices parameters of SD OSCs based on PT2 and Y6 with different D/A thickness. 1% DIO was added into Y6 solution.

Thickness (nm)		V_{oc} (V)	J_{sc} (mA cm^{-2})	FF (%)	PCE (%) (average)[a]
Donor	Accepter				
40	50	0.83	26.2	69.1	15.0 (14.8 ± 0.2)
40	60	0.83	26.5	68.1	15.0 (14.9 ± 0.1)
40	70	0.82	26.4	67.1	14.5 (14.2 ± 0.3)
50	50	0.83	26.7	70.2	15.6 (15.4 ± 0.2)
50	60	0.83	26.7	74.4	16.5 (16.3 ± 0.2)
50	70	0.83	26.5	69.1	15.2 (15.1 ± 0.1)
60	50	0.82	26.3	68.6	14.8 (14.6 ± 0.2)
60	60	0.82	26.1	69.4	14.9 (14.6 ± 0.3)
60	70	0.81	26.4	69.1	14.8 (14.5 ± 0.3)

[a] The average parameters were calculated from 10 independent cells.
[b]EQE values.

Supplementary Table 2. Devices parameters of SD OSCs based on PT2 and Y6 with different DIO contents.

**DIO (%)**	V_{oc} **(V)**	J_{sc} **(mA cm^{-2})**	**FF (%)**	**PCE (%) (average)**[a]
0	0.85	25.1	60.8	13.0 (12.6 ± 0.3)
0.5	0.83	25.9	65.7	14.1 (14.0 ± 0.1)
1	0.83	26.7	74.4	16.5 (16.3 ± 0.2)
1.5	0.81	26.6	69.1	14.9 (14.7 ± 0.2)

[a] The average parameters were calculated from 10 independent cells.

Supplementary Table 3. Devices parameters of BC OSCs based on PT2 and Y6 with different DIO contents.

DIO (%)	V_{oc} (V)	J_{sc} (mA cm^{-2})	FF (%)	PCE (%) (average)[a]
0	0.84	22.6	44.4	8.4 (8.3 ± 0.1)
0.5	0.83	26.3	68.9	15.0 (14.8 ± 0.2)
1.0	0.81	26.1	66.4	14.0 (13.8 ± 0.2)

[a] The average parameters were calculated from 10 independent cells.

Supplementary Table 4. SCLC mobilities of SD and BC devices.

**Device**	**μ_h (10^{-4} cm^2 V^{-1} s^{-1})**	**μ_e (10^{-4} cm^2 V^{-1} s^{-1})**
SD OSC (as-cast)	1.3 ± 0.1	2.3 ± 0.2[a]
SD OSC (optimum)	5.9 ± 0.2	5.4 ± 0.2
BC OSC (optimum)	2.5 ± 0.2	3.5 ± 0.3

[a] The average parameters were calculated from 10 independent cells.

Supplementary Table 5. Summary of device parameters of slot-die printed OSCs with different device area

**Operating Condition**	**V_{oc} (V)**	**J_{sc} (mA cm^{-2})**	**FF (%)**	**PCE (%) (average)[a]**
SD OSC (0.04 cm^2)	0.82	27.3 (26.4)[b]	69.1	15.5 (15.3 ± 0.2)
SD OSC (0.80 cm^2)	0.81	26.4 (25.6)	67.5	14.6 (14.3 ± 0.3)
BC OSC (0.04 cm^2)	0.81	26.6 (26.1)	67.5	14.5 (14.3 ± 0.2)
BC OSC (0.80 cm^2)	0.78	25.6 (25.4)	63.3	12.6 (12.2 ± 0.4)

[a] The average parameters were calculated from 10 independent cells.
[b]EQE values.

Supplementary Table 6. Devices parameters of BC OSCs based on PT2:Y6 by using different PT2 batches. Chlorobenzene was used as host solvent

**Molecular weight (M_n)**	**Operating Condition**	**V_{oc} (V)**	**J_{sc} (mA cm^{-2})**	**FF (%)**	**PCE (%) (average)[a]**
45 kDa	BC	0.78	23.3 (22.1)[b]	63.6	11.6 (11.5 ± 0.1)
57 kDa	BC	0.78	22.5 (22.0)	60.0	10.4 (10.2 ± 0.2)
91 kDa	BC	0.76	22.0 (21.9)	60.0	10.0 (9.9 ± 0.1)

[a] The average parameters were calculated from 10 independent cells.
[b]EQE values.

Supplementary Table 7. Summary of device parameters of the SD OSCs fabricated with PT2 films with CF/DIO solvent washing.

M_n and PDI	V_{oc} (V)	J_{sc} (mA cm^{-2})	FF (%)	PCE (%) (average)[a]
45 kDa (2.33)	0.83	25.8 (25.6)[b]	75.1	16.1 (15.9 ± 0.2)
57 kDa (2.05)	0.82	26.2 (25.9)	74.5	16.3 (16.2 ± 0.1)
91 kDa (2.08)	0.82	26.4 (26.1)	74.0	16.0 (15.8 ± 0.2)

[a] The average parameters were calculated from 10 independent cells.
[b]EQE values.

Supplementary Table 8. Devices parameters of BC OSCs based on PT2:IT-4F by using different PT2 batches. Chloroform was used as host solvent.

**Molecular weight (M_n)**	**V_{oc} (V)**	**J_{sc} (mA cm^{-2})**	**FF (%)**	**PCE (%) (average)[a]**
45 kDa	0.82	20.4 (20.1)[b]	73.2	12.3 (12.1 ± 0.2)
57 kDa	0.81	20.0 (20.1)	67.1	10.8 (10.8 ± 0.0)
91 kDa	0.82	20.3 (20.3)	61.4	10.2 (10.1 ± 0.1)

Supplementary Table 9 Devices parameters of BC OSCs based on PT2:Y6 by using two random PT2 batches. Chloroform was used as host solvent.

Batch Number	Operating Condition	V_{oc} (V)	J_{sc} (mA cm^{-2})	FF (%)	PCE (%) (average)[a]
1	SD	0.83	27.0 (26.3)[b]	73.2	16.3 (16.3 ± 0.0)
	BC	0.82	26.0 (25.5)	71.4	15.3 (15.1 ± 0.2)
2	SD	0.83	27.4 (26.7)	73.7	16.7 (16.4 ± 0.3)
	BC	0.82	26.3 (25.7)	67.2	14.5 (14.4 ± 0.1)

[a] The average parameters were calculated from 10 independent cells.
[b]EQE values.

Supplementary Table 10 Devices carrier mobilities of SD and BC OSCs fabricated with different PT2 batches.

**Blend**	**Operating Condition**	**μ (10^{-5} cm^2 V^{-1} s^{-1})**
PT2 (45k):Y6	SD	10.1
	BC	5.1
PT2 (57k):Y6	SD	10.2
	BC	4.1
PT2 (91k):Y6	SD	7.5
	BC	4.2

Supplementary Reference

1. Liu, T. et al. Optimized Fibril Network Morphology by Precise Side-Chain Engineering to Achieve High-Performance Bulk-Heterojunction Organic Solar Cells. *Adv. Mater.* **30**, 1707353 (2018).
2. Ye, L. et al. Insertion of chlorine atoms onto π-bridges of conjugated polymer enables improved photovoltaic performance. *Nano Energy* **58**, 220-226 (2019).
