# Supplementary material for: Optimized active layer morphology toward efficient and polymer batch insensitive organic solar cells
Source: Nat Commun. 2020 Jun 5;11:2855. doi: 10.1038/s41467-020-16621-x (PMC7275072; doi:10.1038/s41467-020-16621-x)
Supplement: Supplementary file 3 — Reporting Summary [file 41467_2020_16621_MOESM3_ESM.pdf]

nature research

Corresponding author(s): Yanming Sun

Solar Cells Reporting Summary

Nature Research wishes to improve the reproducibility of the work that we publish. This form is intended for publication with all accepted papers reporting the characterization of photovoltaic devices and provides structure for consistency and transparency in reporting. Some list items might not apply to an individual manuscript, but all fields must be completed for clarity.

For further information on Nature Research policies, including our data availability policy, see Authors & Referees.

~ Experimental design

Please check: are the following details reported in the manuscript?

1. Dimensions

Item	Yes/No	Response
Area of the tested solar cells	[x] Yes [ ] No	Section "OSC device fabrication and measurement"
Method used to determine the device area	[x] Yes [ ] No	The electronic active area of the cell is defined by the overlap of the ITO electrode and metal electrode.

2. Current-voltage characterization

Item	Yes/No	Response
Current density-voltage (J-V) plots in both forward and backward direction	[ ] Yes [x] No	Generally, organic photovoltaic devices do not have forward and backward problems. And we only scan the device in forward direction.
Voltage scan conditions *For instance: scan direction, speed, dwell times*	[x] Yes [ ] No	Section "OSC device fabrication and measurement"
Test environment *For instance: characterization temperature, in air or in glove box*	[x] Yes [ ] No	Our devices were characterized at room temperature (ca. 25 Celsius degree) in glove box.
Protocol for preconditioning of the device before its characterization	[ ] Yes [x] No	No preconditioning protocol
Stability of the J-V characteristic *Verified with time evolution of the maximum power point or with the photocurrent at maximum power point; see ref. 7 for details.*	[ ] Yes [x] No	No hysteresis or other unusual behaviour was observed during the characterization of the solar cells. In general, organic solar cells do not have hystereisis problems. We only tested the long-term stability.

3. Hysteresis or any other unusual behaviour

Item	Yes/No	Response
Description of the unusual behaviour observed during the characterization	[ ] Yes [x] No	No hysteresis or other unusual behaviour was observed during the characterization of the solar cells. In general, organic solar cells do not have hysteresis problems.
Related experimental data	[ ] Yes [x] No	No hysteresis or other unusual behaviour was observed during the characterization of the solar cells.

4. Efficiency

Item	Yes/No	Response
External quantum efficiency (EQE) or incident photons to current efficiency (IPCE)	[x] Yes [ ] No	The external quantum efficiency (EQE) spectra of OSCs are recorded using Enli QE-R3011 (Enli Technology Co., Ltd Taiwan).
A comparison between the integrated response under the standard reference spectrum and the response measure under the simulator	[x] Yes [ ] No	There are less than 5% errors between the integrated response under the standard reference spectrum and the response measure under the simulator. We give details in the manuscript.

For tandem solar cells, the bias illumination and bias voltage used for each subcell

- [ ] Yes — *State where this information can be found in the text.*
- [x] No — We only fabricated single junction solar cells.

5. Calibration

Light source and reference cell or sensor used for the characterization

- [x] Yes — Section "OSC device fabrication and measurement"
- [ ] No — *Explain why this information is not reported/not relevant.*

Confirmation that the reference cell was calibrated and certified

- [x] Yes — Section "OSC device fabrication and measurement"
- [ ] No — *Explain why this information is not reported/not relevant.*

Calculation of spectral mismatch between the reference cell and the devices under test

- [x] Yes — The relative expanded uncertainty resulting of the relative combined standard uncertainty multiplied with a coverage factor k = 2 is specified. It corresponds to a level of confidence of 95%.
- [ ] No — We do not have the detailed information for the method.

6. Mask/aperture

Size of the mask/aperture used during testing

- [ ] Yes — *State where this information can be found in the text.*
- [x] No — We did not use a mask during testing and the device effective area was obtained by its crossed area of the bottom and top electrodes.

Variation of the measured short-circuit current density with the mask/aperture area

- [ ] Yes — *State where this information can be found in the text.*
- [x] No — We did not use a mask during testing.

7. Performance certification

Identity of the independent certification laboratory that confirmed the photovoltaic performance

- [x] Yes — The PCE was certified by NIM, China, using a 3.152 mm2 mask.
- [ ] No

A copy of any certificate(s)
Provide in Supplementary Information

- [x] Yes — See "Supplementary Fig. 5".
- [ ] No

8. Statistics

Number of solar cells tested

- [x] Yes — We have tested ten solar cells for each type of OSCs, and the average PCE of OSCs are obtained from ten independent cells.
- [ ] No — *Explain why this information is not reported/not relevant.*

Statistical analysis of the device performance

- [x] Yes — Statistical results of the devices are listed in Table 1,2 and Supplementary Table 1-9.
- [ ] No — *Explain why this information is not reported/not relevant.*

9. Long-term stability analysis

Type of analysis, bias conditions and environmental conditions
For instance: illumination type, temperature, atmosphere humidity, encapsulation method, preconditioning temperature

- [x] Yes — See "Supplementary Figure 6."
- [ ] No — *Explain why this information is not reported/not relevant.*
